# Privacy-Preserving Decision-Tree Evaluation with Low Complexity for Communication

**DOI:** 10.3390/s23052624

**Published:** 2023-02-27

**Authors:** Yidi Hao, Baodong Qin, Yitian Sun

**Affiliations:** School of Cyberspace Security, Xi’an University of Posts and Telecommunications, Xi’an 710121, China

**Keywords:** secure integer comparison, fully homomorphic encryption, decisional tree

## Abstract

Due to the rapid development of machine-learning technology, companies can build complex models to provide prediction or classification services for customers without resources. A large number of related solutions exist to protect the privacy of models and user data. However, these efforts require costly communication and are not resistant to quantum attacks. To solve this problem, we designed a new secure integer-comparison protocol based on fully homomorphic encryption and proposed a client-server classification protocol for decision-tree evaluation based on the secure integer-comparison protocol. Compared to existing work, our classification protocol has a relatively low communication cost and requires only one round of communication with the user to complete the classification task. Moreover, the protocol was built on a fully homomorphic-scheme-based lattice that is resistant to quantum attacks, as opposed to conventional schemes. Finally, we conducted an experimental analysis comparing our protocol with the traditional approach on three datasets. The experimental results showed that the communication cost of our scheme was 20% of the cost of the traditional scheme.

## 1. Introduction

Machine learning is an important part of artificial-intelligence technology, and it can find rules and extract knowledge from large amounts of data and constantly improve itself. The classifiers of machine learning are worthy tools in many scenarios, such as health monitoring, transportation and image recognition [1]. Due to the popularization and development of machine learning, its inherent privacy issues have received widespread concern.

Works on privacy-preserving machine learning are divided into privacy protection in the training stage and privacy protection in the practical stage. Privacy protection in the training stage [2,3,4,5] mainly uses encryption to improve the algorithms in machine learning or to segment datasets, thereby, enhancing the privacy. Privacy protection in the practical phase [6,7,8] mostly uses cryptographic techniques and leverages differential privacy. The latter may not be more efficient but it has better privacy and classification accuracy.

To evaluate data in privacy protection, encryption of the data is required. Moreover, the encrypted data need to support the secondary processing operation to enable reuse of the data. However, the common encryption algorithm often breaks the original algebraic structure of the data, and thus it does not meet this requirement. Homomorphic encryption [9], as a cryptographic technology that can support ciphertext operation and is often applied in privacy protection scenarios of machine learning.

Current mainstream learning classifiers, such as decision trees, Bayesian classifiers and neural networks, require addition, multiplication and other polynomial operations, numerical comparison, probability calculations, Euclidean distance, and other operations as part of their core. Probability operations and Euclidean distance can be transformed into polynomial operations but numerical comparison cannot be directly transformed into a general arithmetic operation, which is the most basic and core operation in all kinds of data analysis, including machine learning.

The simplest numerical comparison operation is the secure comparison problem between two integers. This issue dates back to the original solution to the millionaire problem [10] proposed by Yao in 1982. The operation has become a computational bottleneck to further improving application performance. This has been especially true since the rise of machine learning, as many algorithms involve integer comparison, such as image processing for privacy protection and medical data analysis. Therefore, finding an effective way to solve this problem will have a positive impact on the system’s overall performance.

A typical machine-learning algorithm requires the server to use the data processed by the client to evaluate and return the final classification results; however, the client has the risk of leaking sensitive information at this time. If the client uses the machine-learning model provided by the server for local classification, the complex classification model calculation will also increase the workload of the client and be very time-consuming. Therefore, machine learning in privacy protection technology is necessary.

To protect the privacy of models and user data, many privacy classification schemes have been proposed. The decision-tree-classification model is one of the important classifications used in this field. Existing work uses bit encryption, and the communication cost is relatively high. Moreover, the user needs to interact with the server for many rounds. Therefore, these schemes are not suitable for poor communication environments.

Our work is centered on how to perform private decision-tree evaluation with low communication costs. We designed a protocol based on the client–server model where the server with the decision-tree model provides service to the client when the client needs to classify their private data using the decision-tree model. Ideally, the server cannot infer any information related to the client’s private data, including the final classification results. Further, the client cannot obtain any information about the classification decision tree other than the final classification results.

### 1.1. Our Contributions

In this work, we design and implement a secure integer-comparison method and a novel privacy-preserving decision-tree-classification scheme combined with the linear evaluation methods of Tai et al. [11]. The idea mainly uses lattice-based, fully homomorphic encryption [12], allowing the server to run the complete private decision-tree-classification process. As a result, we provide an integer-comparison protocol with no interaction based on a new secure integer-comparison method combined with [13].

During the protocol, the client simply encrypts their own private feature vector, sends it and then waits to receive the ciphertext with the classification result from the server. The entire decision-tree-evaluation process is delegated to the server. This delegation not only reduces the computational burden on the client but also greatly reduces the communication costs. The main contributions of our work are summarized below:

- We propose a secure integer-comparison algorithm that can resist quantum attacks. The security of the comparison algorithm is improved using a fully homomorphic encryption system based on lattice problems. Compared with classical comparison methods, such as DGK, the computation depth is only $\log_{2}m$ instead of *m* for *m*-bit integers.
- We creatively provide a protocol with no interaction that allows the server to complete the decision-tree-classification process after the client sends encrypted privacy data. At the end, the client obtains the final classification result by decrypting the received ciphertext from the server. Therefore, our protocol has low communication costs and very simple client operations of encryption and decryption.
- Finally, we implement the protocol using the SEAL library and perform a series of experiments on real datasets from the UCI repository. The experimental results show that our bandwidth is no more than 20% of that of Tai et al.’s protocol.

### 1.2. Related Work

In 2007, Brikell [14] combined homomorphic encryption with garbled circuits to propose the first private decision-tree-evaluation protocol. However, the communication cost of this protocol is linear and not suitable for large decision trees. Bost et al. [15] used fully homomorphic encryption to design an evaluation scheme of a decision tree for privacy protection in 2014. In the decision evaluation, the decision tree was treated as a high-order polynomial, and the classification result of this decision tree could be obtained directly by computing this polynomial.

Although the scheme protects the private data owned by the user, it has many problems. In addition to leaking information about the decision-tree model, it has a complex and inefficient computation process. In 2016, Wu et al. [16] used oblivious transfer technology [17] and additive homomorphic encryption to replace fully homomorphic encryption, reducing the computational cost. However, this scheme requires the client to obtain the complete decision tree randomized by the server; therefore, its communication cost grows exponentially with the depth of the decision tree.

In 2017, Tai et al. [11] used Damgård’s secure integer-comparison scheme [18] (shorted as DGK) and expressed the decision-tree machine-learning model as a linear function. In Tai’s scheme, the final classification node is determined by calculating the path cost of the nodes in the decision tree. The scheme avoids multiplication between encrypted messages, and the client does not need to receive the complete decision tree after randomization by the server. Compared with other schemes, it has good efficiency.

The efficiency of Tai’s scheme is, in part, due to the Lifted ElGamal [19] based on Elliptic Curve Cryptography—a cryptographic scheme with faster homomorphic operations and smaller ciphertexts. The downside is that it is not resistant to quantum attacks. Their protocol needs multi-round interactions, and the communication cost grows linearly with the number of decision tree nodes. Thus, Tai’s scheme is not suitable for an environment with poor communication. In addition, the client takes on heavy computing tasks and needs to cooperate with the server to calculate the intermediate results but only obtains the final classification results.

In 2018 Lu et al. [20] proposed a non-interactive comparison protocol based on the BGV [21] homomorphic encryption scheme. This scheme has a low multiplication depth and high efficiency. However, it is only suitable for small inputs and has poor scalability. In addition, the output length of the server is exponentially related to the depth of the tree. Tueno [22] represented the decision tree as an array for traversal. Kiss [23] solved privacy concerns by dividing multiple subfunctions into modules. Ma et al. [24] proposed a secure comparison protocol that only requires a sublinear quantity in 2011. Bai et al. [25] designed a scheme based on shared oblivious selection combined with a tree-encoding approach in 2022. The scheme reduced the number of decision nodes to run the comparison protocol.

In 2022, Veugen [26] proposed a lightweight secure integer-comparison scheme. The communication cost of this scheme is low but the communication cycles are equal to the number of input bits. The communication cost in these private decision-tree-evaluation schemes is relative to the nodes number of the tree and requires the client to have great computational power for the decision-tree evaluation. These existing schemes require many interaction rounds and have high communication costs. We further compare the performance of these schemes theoretically in the performance analysis section of this article.

### 1.3. Organization

The rest of this paper is organized as follows: we review the background knowledge about fully homomorphic encryption and decision-tree classifiers in Section 2. A detailed description of the proposed secure integer comparison can be found in Section 3. We describe the calculation of the path cost and the evaluation of the privacy decision tree in Section 4. The implementation and evaluation details are given in Section 5, including the analysis of the experimental and theoretical data. Finally, the conclusion of this paper is found in Section 6.

## 2. Preliminary

### 2.1. Fully Homomorphic Encryption

A fully homomorphic encryption scheme (abbreviated as FHE) that can be conducted on ciphertext additions and make multiplication feasible is illustrated. Compared with the traditional encryption method, FHE pays more attention to the security of data processing. Data processing refers to calculation by a function consisting of addition and multiplication. Security means that no information of the plaintext will be leaked while processing the encrypted data, and the decryption will be equal to the plaintext after processing. Next, we introduce the encryption algorithm adopted in this paper and related basic algebraic knowledge.

**Definition 1**  (Polynomial Ring)**.** *For the polynomial $f\left(x\right)=a_{n-1}x^{n-1}+a_{n-2}x^{n-2}+\ldots +a_{0}$ where its coefficients belong to a ring*. *The set of the polynomial is called a polynomial ring denoted R=Z[x]/f(x)*. *For the polynomial ring that we usually use in cryptography, the polynomial f(x)∈Z[x] is a monic irreducible polynomial*. *To facilitate study, the most common setting is to take $f\left(x\right)=x^{d}+1$ and the integer $d=2^{s}$*, *where s is a positive integer*.

**Definition 2**  (RLWE Distribution [27])**.** *Given a field tensor product K and the integer module q=q(λ)≥2*, where λ is the security parameter and $R^{*}$ indicates the dual fractional ideal of R, *then $R_{q}$ is the polynomial coefficient on this ring and is not greater than q*, and χ denotes an error distribution over K. For the secret vector $s\in R_{q}^{*}$, *the ring learning with errors (abbreviated as RLWE) distribution ${\mathrm{LWE}}_{s,\chi }$ can be obtained by uniformly choosing a random element a from $R_{q}$ and the noise term e∈χ and outputting (a,b)*, where $b=\left(a\cdot s\right)/q+e\;mod\;R^{*}$.

**Definition 3**  (Decision RLWE [27])**.** *The decision RLWE problem is an extensive version of the RLWE problem*. *This problem is to determine whether a vector v belongs to a uniform distribution or a RLWE distribution*.

Next, we can introduce the BFV-encryption scheme [12] based on the decision RlWE problem. The plaintext space of the BFV-encryption scheme is represented as $R_{t}$, where *t* is a positive integer. We obtained the expression $q=\Delta \cdot t+r_{t}\left(q\right)$ from the settings Δ=⌊q/t⌋ and $r_{t}\left(q\right)=q\;\mathrm{mod}\;t$, where the details of the integer *q* are in the decision-RLWE definition. Specifically, the BFV scheme includes the following four algorithms:

- BFV-KeyGen: First, set sample $s\leftarrow R_{2}$ as the private key SK. Then, choose *a* from the ring $R_{q}$ and noise term *e* from the distribution χ uniformly at random and obtain the public key $PK=({\left[-\left(a\cdot s+e\right)\right]}_{q},a)$. In addition, the BFV-encryption scheme requires a special key, called a relinearization key, which is mainly used to cooperate with homomorphic multiplication. Sample $a_{0}\leftarrow R_{p\cdot q}$, $e\leftarrow \chi^{\prime }$ (simply taking $\chi =\chi^{\prime }$ will result in a lost security). For real k>0 and constant α, if we assume ∥χ∥<B and $p\cdot q=q^{k}$, a relation that $∥\chi^{\prime }∥=B_{k}>\alpha^{1-\sqrt{k}}\cdot q^{k-\sqrt{k}}\cdot B^{\sqrt{k}}$ will limit the distribution $\chi^{\prime }$. Finally, this gives the relinearization key $RLK=({\left[-\left(a\cdot s+e\right)+p\cdot s^{2}\right]}_{p\cdot q},a)$.
- BFV-Enc: to encrypt a plain message *m*, set $P_{0}=PK\left[0\right]$, $P_{1}=PK\left[1\right]$, choose $u\in R_{2}$ and $e_{1},e_{2}\in \chi$ uniformly at random and return $ct=({\left[P_{0}\cdot u+e_{1}+\Delta \cdot m\right]}_{q},{\left[P_{1}\cdot u+e_{2}\right]}_{q})$.
- BFV-Dec: Set $c_{0}=ct\left[0\right]$, $c_{1}=ct\left[1\right]$ and calculate ${\left[\left\lfloor t\cdot {\left[c_{0}+c_{1}\cdot s\right]}_{q}/q\right\rceil \right]}_{t}$.
- BFV-Eva: The basic homomorphic operations are addition and multiplication. Simply compute $\left({\left[ct_{1}\left[0\right]+ct_{2}\left[0\right]\right]}_{q},{\left[ct_{1}\left[1\right]+ct_{2}\left[1\right]\right]}_{q}\right)$ for addition between ciphertexts $ct_{1}$ and $ct_{2}$.
  Homomorphic multiplication is relatively complicated. Compute
  (1) $$\begin{array}{ccc} c_{1,0} & = & {\left[\left\lfloor t\cdot (ct_{1}\left[0\right]\cdot ct_{2}\left[0\right])/q\right\rceil \right]}_{q} \end{array}$$
  (2) $$\begin{array}{ccc} c_{1,1} & = & {\left[\left\lfloor t\cdot (ct_{1}\left[0\right]\cdot ct_{2}\left[1\right]+ct_{1}\left[1\right]\cdot ct_{2}\left[0\right])/q\right\rceil \right]}_{q} \end{array}$$
  (3) $$\begin{array}{ccc} c_{1,2} & = & {\left[\left\lfloor t\cdot (ct_{1}\left[1\right]\cdot ct_{2}\left[1\right])/q\right\rceil \right]}_{q} \end{array}$$
  The multiplication of the ciphertext increases the ciphertext dimension, which makes homomorphic calculation more complicated and increases the decryption difficulty. It uses the relinearization key RLK to reduce the ciphertext dimensions. Compute
  (4) $$\left(c_{2,0},c_{2,1}\right)=\left({\left[\left\lfloor c_{1,2}\cdot RLK\left[0\right]/p\right\rceil \right]}_{q},{\left[\left\lfloor c_{1,2}\cdot RLK\left[1\right]/p\right\rceil \right]}_{q}\right)$$
  and return $\left({\left[c_{1,0}+c_{2,0}\right]}_{q},{\left[c_{1,1}+c_{2,1}\right]}_{q}\right)$.

### 2.2. Decision-Tree Classifiers

The input of the user is a feature vector whose dimensional is *n*, $x=\left(x_{1},\ldots ,x_{n}\right)\in Z^{n}$. The function of a decision-tree classifier is to provide classification services to users by processing the input of the users. Without loss of generality, we assume that the decision tree is a full binary tree. There are *m* non-leaf nodes and m+1 leaf nodes in a full binary tree. Let *T* be the evaluation function of the decision tree, and the final classification result of the input *x* is the output v=T(x).

The input of the user is tested at each non-leaf node in the tree. The root of the decision tree is the starting point for evaluation. The classification result is judged to be in the left branch or the right branch of that node based on the test of the current decision node (not a leaf node). Continue testing at the left child or right child of the current node. Loop this process until a leaf node is reached and the category to which the input *x* belongs is obtained.

## 3. Integer Comparison Protocol

Our secure integer-comparison protocol is based on the method of integer comparison from [13]. Given two integers, *x* and *y*, we decompose them in binary form $x=\sum_{i=0}^{m}x_{i}2^{i}$, $y=\sum_{i=0}^{m}y_{i}2^{i}$ and obtain two sequences $\left(x_{0},\ldots ,x_{m}\right)$, $\left(y_{0},\ldots ,y_{m}\right)$, where $m=\max(\log_{2}x,\log_{2}y)$. We can use the idea of a substring to solve the integer comparison and finally obtain the comparison result x>y. (The comparison result between integers *x* and *y* is a boolean. If *x* is greater than *y*, the result is one; otherwise, it is zero.)

There are two integers, *x* and *y*, that have been binarily decomposed, and we split the binary sequence *X* and *Y* of the two integers into two substrings: $X=X_{sub1}X_{sub0}$ and $Y=Y_{sub1}Y_{sub0}$. In addition, the corresponding substrings have the same length, such as $X_{sub1}$ and $Y_{sub1}$ sequences have the same length. Then, we can obtain

(5) $$\left(x>y\right)=\left\{\begin{array}{cc} X_{sub1}>Y_{sub1} & X_{sub1}\neq Y_{sub1}\\ X_{sub0}>Y_{sub0} & X_{sub1}=Y_{sub1} \end{array}\right.$$

This can also be expressed as

(6) $$\left(x>y\right)=\left(X_{sub1}=Y_{sub1}\right)\left(X_{sub0}>Y_{sub0}\right)+\left(X_{sub1}>Y_{sub1}\right)$$

In other words, it can partition the two binary sequences *X* and *Y* corresponding to integers *x* and *y* into two substrings of equal length and output the result of comparing the two integers according to the magnitude relationship of the decimal integers represented by the subsequence. We use $eq_{i,j}$ to indicate whether two binary substrings of length *j* starting with the *i*-th bit are equal. More intuitive, $eq_{i,j}$ is a boolean that stands for the relation of = between the two binary strings $x_{i+j-1},x_{i+j-2},\ldots ,x_{i}$ and $y_{i+j-1},y_{i+j-2},\ldots ,y_{i}$. For a substring that only has one bit, we can intuitively compute $eq_{i,1}=1-{\left(x_{i}-y_{i}\right)}^{2}$. Then, we obtain

(7) $$eq_{i,j}=\left\{\begin{array}{cc} 1-x_{i}+2x_{i}y_{i}-y_{i} & j=1\\ eq_{i+l,j-l}eq_{i,l} & j>1 \end{array}\right.$$

In this formula, the integer *l* is the partition index of the binary sequence and 0<l<j. Next, we use $eq_{i,j}$ to represent $c_{i,j}$, where the $c_{i,j}$ is the result of comparing two binary strings of length *j* starting with the *i*-th bit.

(8) $$c_{i,j}=\left\{\begin{array}{cc} x_{i}-x_{i}y_{i} & j=1\\ eq_{i+l,j-l}c_{i,l}+c_{i+l,j-l} & j>1 \end{array}\right.$$

In addition, when the partition index *l* satisfies l≈⌊j/2⌋[13], the recursion depth in the calculation is logarithmically related to the input original binary sequence length *m*, which is the optimal depth.

The process of comparison essentially divides the binary sequence of integers into two fractions. Until the substring length is 1 bit, the backtracking begins and returns the comparison result of the corresponding substring. Then, we can use the recursive formula to calculate the substring comparison with the double length and continue recursively to obtain the final comparison result of the two integers.

We designed a secure comparison protocol to obtain the comparison result of two encrypted inputs. Decompose two integers, *x* and *y*, into binary bits of equal length. We obtain the binary strings $\left\{x_{0},x_{1},\ldots ,x_{t-1}\right\}$ and $\left\{y_{0},y_{1},\ldots ,y_{t-1}\right\}$, where each integer is less $2^{t}$. Use the BFV-encryption scheme to encrypt and obtain $\left\{\left[x_{0}\right],\left[x_{1}\right],\ldots ,\left[x_{t-1}\right]\right\}$. It is important to note that not only is the input processed in ciphertext form but the final comparison result is also encrypted.

In the comparison process, the bit sequences *x* and *y* are divided into $X_{1}$, $X_{2}$ and $Y_{1}$, $Y_{2}$ of equal length until the length of the subsequence is 1; then, the size comparison between substrings is equivalent to the comparison between bits. Finally, the output generated by the comparison between the substrings is combined to obtain the final comparison result.

This comparison method is most efficient when the relationship between the partition index *l* and the length *j* in the formula is l≈⌊j/2⌋. Thus, we set l≈⌊j/2⌋ in the algorithms as follows. The comparison algorithm has a detailed description as shown in Algorithm 1.
**Algorithm 1**CMP([x],y): Compare the encrypted *x* and integer *y*
**Input:** The ciphertext string $\left\{\left[x_{0}\right],\ldots ,\left[x_{t}-1\right]\right\}$ obtained by bitwise encryption of the integer *x*, where $x=\sum_{i=0}^{t-1}x_{i}2^{i}$ and an integer *y*.**Output:** Comparison result in ciphertex between integer *x* and *y*. 1:Binary decomposition of the integer *y*, $y=\sum_{i=0}^{t-1}y_{i}2^{i}$ 2:Recursively compute $Z_{i,j}$ and $t_{i,j}$ as the following formula:
$$eq_{i,j}=\left\{\begin{array}{cc} 1-x_{i}+2x_{i}y_{i}-y_{i} & j=1\\ eq_{i+l,j-l}eq_{i,l} & j>1 \end{array}\right.$$
$$c_{i,j}=\left\{\begin{array}{cc} x_{i}-x_{i}y_{i} & j=1\\ eq_{i+l,j-l}c_{i,l}+c_{i+l,j-l} & j>1 \end{array}\right.$$ 3:Return comparison result $c_{0,t}=\left[x>y\right]$.

Today, computers are typically multi-core processors, which are designed to increase the computing power by allowing multiple tasks to run simultaneously. Our comparison scheme is not like the previous comparison strategy, which requires the calculation order in serial. It can be computed in parallel and is suitable for multiprocessor environments.

We can set an integer $t_{0}$ as the maximum single comparison length. When the bit lengths of secret numbers are lower than $t_{0}$, we directly use a single processor for secure integer comparison. If the bit length is greater than $t_{0}$ bits, we can use multiprocessor secret number comparisons. This approach reduces the load of a single processor and improves the overall comparison efficiency.

Using the comparison scheme given in the previous section, we can convert the comparison of two integers into an operation between bits and finally obtain the comparison result of two ciphertexts.

**Figure 1:**
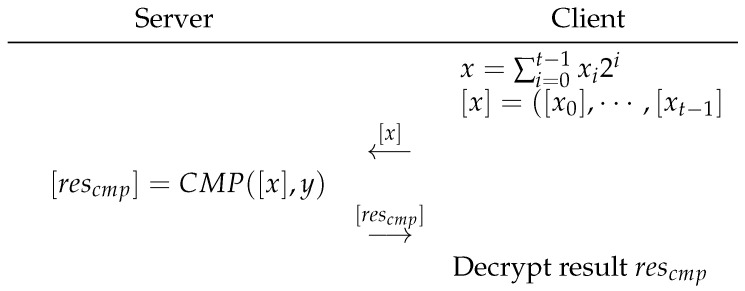
Integer-comparison protocol.

The interaction protocol has two parties, client and server. The client has the secret integer *x*, and the server has the secret integer *y*. In addition, the client holds the private key of the BFV encryption. The public key is public information for both the client and the server. The interaction protocol of secure integer comparison is shown in Figure 1:

The interaction at each step is as follows:

1. Client: Binarily decomposes the data it holds and sends the corresponding ciphertext $\left\{\left[x_{0}\right],\left[x_{1}\right],\ldots \left[x_{t-1}\right]\right\}$ to the server.
2. Server: The interactive protocol server does not need to encrypt the integers it holds into bits. It is more efficient to use the CMP algorithm directly by the basic operation between plaintext and ciphertext that BFV encryption allows. Furthermore, the result generated after running CMP is in cipher text form, which will not disclose the data held by the server. After the calculation, the server returns the comparison result $res_{cmp}$ in the ciphertext form to the client.
3. Client: The client decrypts the received data directly and obtains the comparison result.

The above is a secure integer-comparison interaction process. In addition, the noise contained in the ciphertext increases with the number of homomorphic operations. A higher upper limit is required for the encryption scheme to be able to accommodate noise. However, this makes the homomorphism calculation take a longer time.

## 4. Private Decision-Tree-Classification Protocol

**Figure 2:**
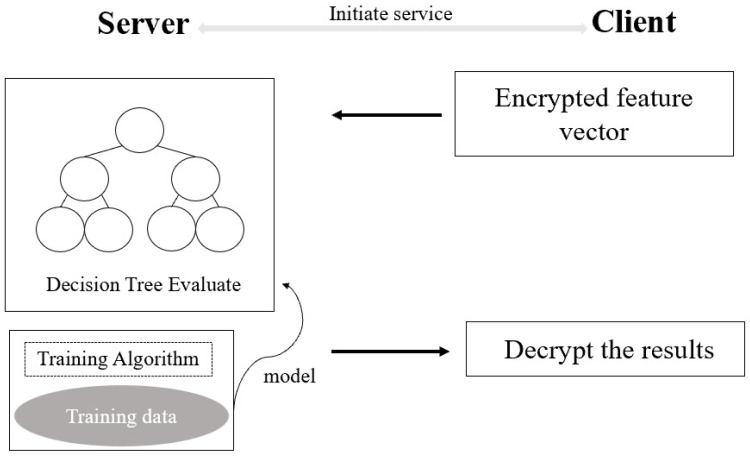
Architecture of private decision-tree classification.

In Section 3, we described the interaction process of how to compare two integers securely. In this section, we propose a new private decision-tree-evaluation scheme based on the secure integer-comparison protocol described above. Here, we give the architecture in Figure 2 for the private decision-tree classification.

### 4.1. Secure Path Evaluation

The path judgment, in essence, aims to compare two feature vectors at each decision node. We assume that an eigenvector in vector space is n-dimensional, denoted as $\left(x_{1},\ldots x_{n}\right)$. We use $x_{i,j}$ to represent the binary bit of each component in the eigenvector. In the classification process, we need the comparison result between the threshold that the node holds and the private input of the client at each decision node using the CMP algorithm. These comparison results are combined with the path cost and edge cost in the decision tree to obtain the final classification output.

**Figure 3:**
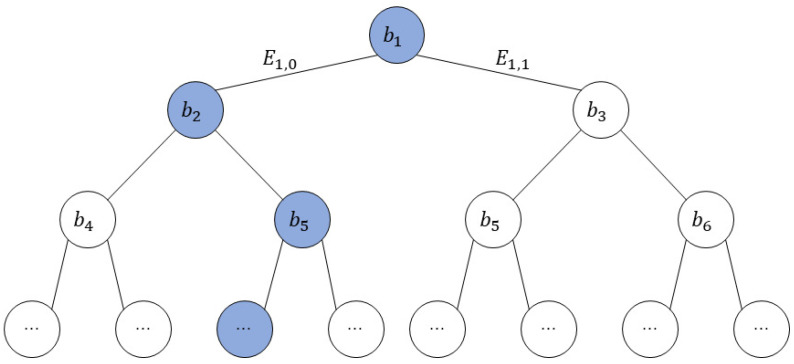
Decision path of the classification result.

Assuming that the decision tree has *m* decision nodes, the server obtains the result $b_{i}$ of the comparison of each decision node $D_{i}$, where i={1,…,m}. The Boolean value $b_{i}=0$ means that the classification result is in the left subtree of the current decision node $D_{i}$; otherwise, it is in the right subtree. Suppose $b_{1}=0,b_{2}=1,b_{5}=0\ldots$; the decision path is shown in blue in Figure 3:

Every decision node $D_{i}$ has a left (right) output edge $E_{\left(i,0\right)}\left(E_{\left(i,1\right)}\right)$ that contains the decision result. Every leaf node $L_{k}$ that represents a classification category has only one path. The path starts at the root node of the decision tree, where k∈{1,…,m+1}. The path is denoted as $P_{k}$, which is essentially the set of edges on that path. Define the cost of each edge $E_{i,j}$ as $e_{i,j}$, where j=0 is the left edge of the current node; otherwise, it is the right edge.

The path cost $pe_{k}$ is obtained by adding up the costs of the edge on this path $P_{k}$, such as the path cost $pe_{3}=e_{1,0}+e_{2,1}+\ldots$ of the third leaf node in Figure 3. We set the edge cost $e_{i,j}$ according to the comparison result $b_{i}$ of each non-leaf node as $e_{i,0}=b_{i}$ and $e_{i,1}=1-b_{i}$. Then, we can further calculate the path cost $pe_{3}=b_{1}+\left(1-b_{2}\right)+\ldots$. When the path cost of a leaf node is zero, the category represented by the node is the classification result.

Based on the above evaluation mechanism, the server can use the ciphertext $\left[b_{k}\right]$ to obtain the path cost in ciphertext form of all leaf nodes. The server sums up the costs corresponding to each edge in the set $P_{k}$ of the paths of each leaf node to obtain the cost of each leaf node path. In this way, the calculation of the path cost is equivalent to the calculation of a linear function at each leaf node. It can be determined which node is the final classification result by determining whether the path cost of each leaf node is zero. The client does not need to know information about the decision tree or interact with the server during the decision-tree-evaluation process, which reduces the communication costs of the solution.

### 4.2. Secure-Classification Generation

In the path evaluation, the server can calculate the path cost of the leaf nodes by setting the cost of each edge. Next, the server adds the classification value $v_{k}$ held by the k-th leaf node to the path cost corresponding to that node. Using the value $v_{k}$, the client can obtain the category that the feature vector it holds belongs to in order to hide the threshold held by each node and prevent the client from inferring some information related to the decision tree of the received data. Thus, the server needs a randomization operation.

After secure path evaluation, the server obtains the ciphertext of the path cost of the leaf node of the decision tree. Next, the server outputs the ciphertext of the corresponding classification results of the leaf node and sends the result of path cost randomization to the client. The client decrypts the ciphertext by checking whether the path cost $pe_{k}$ sent by the server is zero or not, thus, obtaining the corresponding final classification result.

**Figure 4:**
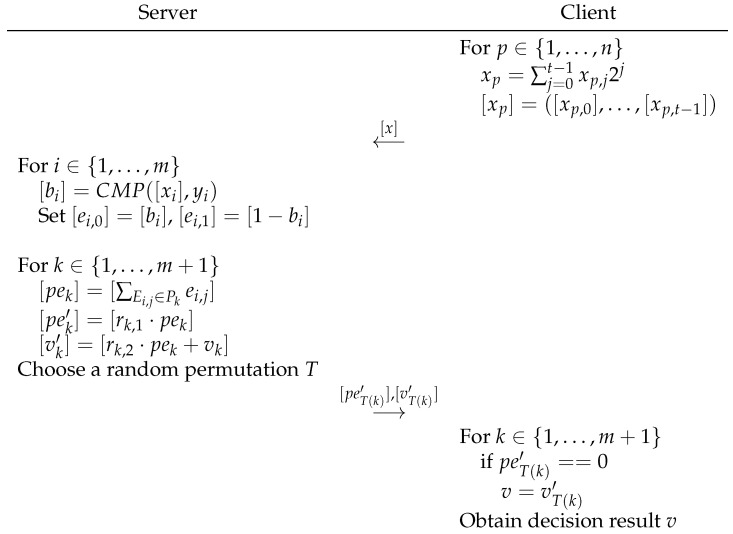
Private decision-tree-classification protocol.

We assume that the dimension of the eigenvector is *n*. Each component of the vector for the client and server is represented by $x_{i}$ and $y_{i}$. The decision tree held by the server has *m* decision nodes, m+1 leaf nodes and uses $L_{k}=\left\{L_{1},L_{2},\ldots ,L_{m+1}\right\}$, which represents a set of leaf nodes. The interaction process is shown in Figure 4.

1. Client: Encrypt their own held feature vector by bit. Send the ciphertext to the server.
2. Server: For every non-leaf node $D_{i}$, where i∈{1,…m}, run the secure comparison algorithm CMP between the threshold of each decision node $D_{i}$ and $x_{i}$ sent by the client. In addition, set the edge cost of the node as $e_{i,0}=b_{i}$ for the edge $E_{i,0}$ and $e_{i,1}=1-b_{i}$ for the edge $E_{i,1}$. For each leaf node $L_{k}$, where k∈{1,…m+1}, calculate the cost of the path by $pe_{k}=\Sigma_{E_{i,j}\in P_{k}}e_{i,j}$. Choose two random integers $r_{k,1}$ and $r_{k,2}$. In addition, compute the randomized path cost $\left[pe_{k}^{\prime }\right]=\left[r_{k,1}\cdot pe_{k}\right]$, $\left[v_{k}^{\prime }\right]=\left[r_{k,2}\cdot pe_{k}+v_{k}\right]$. After $\left[pe_{k}^{\prime }\right]$ and $\left[v_{k}^{\prime }\right]$ are calculated for each leaf node, choose a random permutation *T* over {1,…,m+1} and compute $\left[{pe}_{T(k)}^{\prime }\right]$ and $\left[v_{T(k)}^{\prime }\right]$, where k∈{1,…m+1}, and return them to the client.
3. Client: Decrypt $\left[v_{T(k)}^{\prime }\right]$ and output $v=v_{T(k)}^{\prime }$, if and only if ${pe}_{T(k)}^{\prime }=0$.

The final decision-tree-classification result is obtained by the client if both the client and server follow the protocol. The correctness mainly depends on whether the server can evaluate the correct classification path of each node. Respectively, set the edge cost as $e_{i,0}=b_{i}$ and $e_{i,1}=1-b_{i}$. When the comparison result of a decision node is 0(or1), it will enter the left (or right) branch of the current node.

According to the definition of classification path $pe_{k}=\Sigma_{E_{i,j}\in P_{k}}e_{i,j}$, we have $pe_{k}=0$ under the condition of $e_{i,j}=0$ corresponding to $\forall E_{i,j}\in P_{k}$. After randomization $pe_{k}^{\prime }=r_{k,1}\cdot pe_{k}=0$, we have the decision result $v_{k}^{\prime }=r_{k,2}\cdot pe_{k}+v_{k}=0+v_{k}=v_{k}$. Therefore, the protocol shown in Figure 4 is correct. In terms of security, our scheme is based on the BFV-encryption scheme. There are four main ways to implement post-quantum cryptographic algorithms: hash-based, code-based, multivariable-based and lattice-based. BFV encryption is based on the difficult problems of the lattice-based algorithm, which is resistant to quantum attacks.

### 4.3. Random Forest Expansion

Random forest is a classifier that uses multiple trees to train and predict samples through the idea of ensemble learning. It obtains the final decision result using the classification results of multiple decision trees and solves decision trees’ shortcoming of weak generalization.

Suppose this random forest consists of N decision trees, then, after inputting samples, N-classified results are generated. Generating a classification result from a decision tree is considered as voting for that classification. The server processes the classification results of each decision tree according to the voting mechanism, among which, the most commonly used voting mechanism is majority rule (the classification with the highest number of votes is the final decision result). We can apply our construction to the random forest. We can add a few details based on the interaction protocol as shown in Figure 4. After receiving input from the client, the server adds it into each decision tree for evaluation.

**Figure 5:**
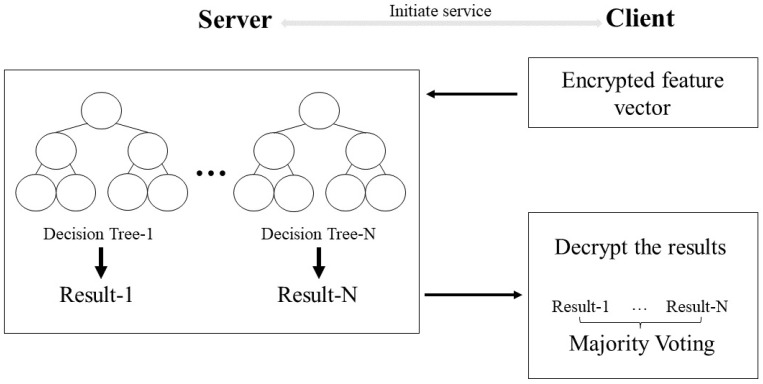
Random forest.

In particular, the server needs to send all classification results of each decision tree in Step 2 of the protocol. The client side process the output of each decision tree to obtain the random forest classification result in Step 3. Specifically, the classification results of a single decision tree can be obtained by judging whether the path cost of the leaf node is zero in each decision tree. The classification with the highest occurrence time is used as the prediction result of the random forest as shown in Figure 5:

## 5. Performance Analysis

### 5.1. Theoretical Analysis

In this section, we discuss the relevant complexity theoretically. For secure integer comparison, the client needs to decompose each element of the eigenvector to a binary string, then encrypt the binary strings and send them to the server. We assume that the feature vector is *n*-dimensional, the feature component size is at most *t* bits, and there are *m* decision nodes and m+1 leaf nodes in the binary decision tree. Thus, the client side generates n·t ciphertexts and send them to the other side. The server obtains the comparison results for each decision node by running the comparison protocol and setting the edge cost *E*; calculating the path cost $pe_{k}$ and the decision result $v_{k}$ based on the edge cost, where k∈{1,…,m+1}; randomizing them; and returning them to the client. Thus, the server only needs to send the path cost and the decision result to the client during the protocol.

In Table 1, $CNumber_{c}$ and $CNumber_{s}$ indicate the quantity of ciphertexts sent by the client and server, respectively. Let us briefly analyze the efficiency of the comparison method. Tai et al.’s protocol required bringing each bit of a single feature value into the formula in a sequential manner. There is linear relativity between the complexity of the calculation and the bits of the feature vector. Our protocol is a dichotomous idea. There is a logarithmic relationship between the complexity of the calculation and the bits of the eigenvector. In addition, the operations required by the client in our protocol are much simpler than those in Tai et al.’s protocol, requiring only one round of interaction.

In Table 2, we compare our private decision-tree-classification protocol with other related works. The communication cost in our protocol is linearly related to the leaf node quantity, while the other protocols are exponentially related to the height of the tree. In addition, the interaction rounds of our protocol as well as [20] require only one round during the whole protocol.

### 5.2. Simulation

To further analyze the performance of our secure integer-comparison scheme in Algorithm 1, we implemented it and compared it with Tai et al.’s integer-comparison scheme. Our algorithm was implemented with the BFV-encryption scheme provided in the SEAL library (https://github.com/microsoft/SEAL, accessed on 1 December 2022). SEAL is a homomorphic encryption library that allows additions and multiplications to be performed on encrypted integers or real numbers.

In addition, it is easy to compile and run in many different environments. We also implemented the integer-comparison scheme proposed by Tai et al. to restore it as much as possible and used the lifted ElGamal scheme implemented by the MCL library mentioned in Tai et al.’s work. MCL is a library for pairing-based cryptography that supports optimal Ate pairing over BN curves and BLS12-381 curves.

The hardware environment of the schemes was an Intel (R) Core (TM) i7-7500U CPU @2.20 GHz processor with 8 GB RAM. The operating system was RedHat 8, and the decision tree of these databases was trained by Sklearn. The programming language was C++, and the development tool of the experimental program was a gcc compiler collection. The data in the table are the average of 10 experiments.

Before analyzing the schemes, we collected the experimental data of the basic operation of BFV encryption and lifed-ElGamal encryption. BFV encryption is an encryption scheme defined over a ring. We set the parameter to provide an encryption key with a security level of at least 128 bits in our experiment. Table 3 summarizes the parameters of the encryption key and ciphertext in addition to the times needed to perform encryption and decryption and related calculations.

It is shown in Table 3 that the BFV encryption, compared with Lifted-ElGamal, has lower efficiency but a smaller ciphertext size, which further reduces the bandwidth cost of our private decision-tree-evaluation protocol.

**Figure 6:**
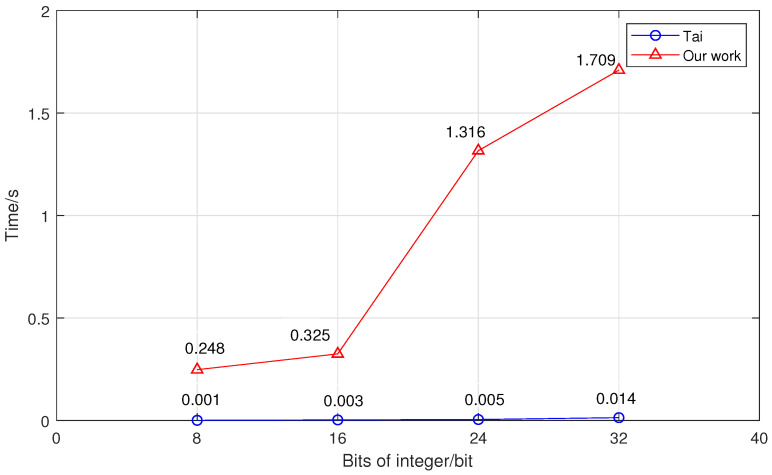
The relationship between the runtime and integer size [11].

**Figure 7:**
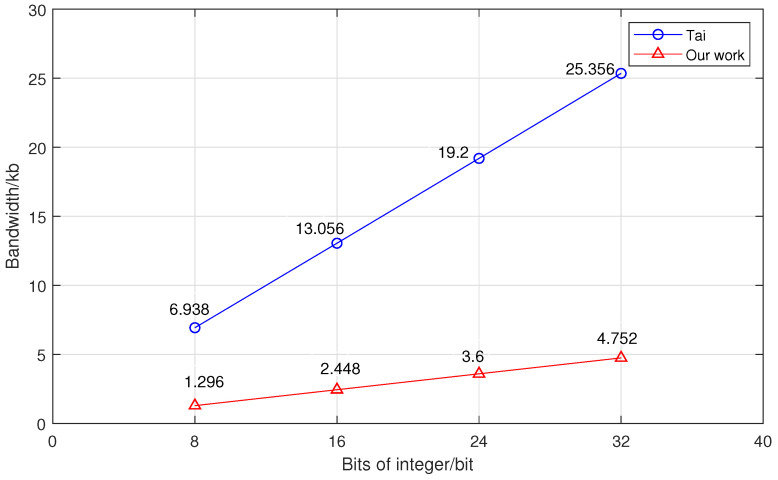
The relationship between the bandwidth and integer size [11].

Next, we compared the secure integer-comparison scheme with Tai et al.’s scheme, and the experimental data can be seen in Figure 6 and Figure 7. We analyzed the performance of the secure integer-comparison protocol in terms of both the runtime and bandwidth. The values of both time and bandwidth for comparison became larger as the size of the integer increased. Due to the performance of BFV encryption, our solution had slightly more runtime compared to Tai’s scheme. Nevertheless, it required less than 2 s to compare two 32-bit integers. In terms of communication costs, our scheme reduced the bandwidth to no more than 20% of Tai et al.’s scheme. For example, for 32-bit integers, the bandwidth of our scheme was only 4.752 kb, while Tai et al.’s scheme required 25.356 kb.

Table 4 shows the runtime and bandwidth of our private decision-tree-classification protocol in various real datasets. We used three databases originating from UCL for the experiment: the Heart Disease dataset, Breast Cancer Wisconsin dataset and Spambase data. The complexity of the decision trees trained on these databases increased. The basic information of the decision tree generated by training is shown in the table. We used it to test the computation cost and communication cost of our scheme and Tai et al.’s scheme.

From the experimental results, we concluded that the more complex the decision tree, the higher the time and bandwidth costs for the two schemes. Part of the reason for the low runtime of Tai is the Lifted-ElGamal encryption based on Elliptic Curve Cryptography, which allows for a fast runtime. However, this encryption system has one drawback: it cannot resist quantum attacks. The lattice-based BFV encryption adopted in our scheme solves this problem and has higher security.

On the other hand, our protocol had a better communication bandwidth. The bandwidth was reduced to about 20% of Tai. et al.’s protocol. Even for Spambase data, which had the most complex decision tree with the largest number of nodes, the bandwidth of ours was about 0.26 MB, while Tai et al.’s protocol was 1.53 MB. Additionally, the rounds of interactions were reduced to only one round in our protocol, making the protocol more friendly for the client.

## 6. Conclusions

In this paper, we proposed a secure integer-comparison scheme based on fully homomorphic encryption. The binary string of integers was divided into substrings to complete the secure comparison of integers. Based on the secure integer-comparison scheme, we designed a private decision-tree-classification model. After obtaining the path cost and path result of each leaf node using the model, the path result was the classification result if the path cost of the node was zero. This evaluation method avoids sending a complete decision tree to the client. In our scheme, the complexity increases linearly with the quantity of decision nodes rather than with the quantity of all nodes in the tree. This is more suitable for decision-tree models in real life, which are usually high and sparse.

At the same time, the scheme simplified client operations, and only one round of interaction was needed between the server and the client, which further reduced the communication cost in the model. In addition, the application of fully homomorphic encryption made the scheme resistant to quantum attacks and improved the security of the protocol. However, our protocol had weak efficiency in terms of its runtime. In follow-up work, we will mainly study how to improve the efficiency of our private decision-tree-classification protocol while reducing communication costs and maintaining quantum-resistant security.

## Figures and Tables

**Table 1:** Communication complexity at the side of the client and server.

Communication	Tai	This Work
$CNumber_{c}$	O((n+m)·t)	O(n·t)
$CNumber_{s}$	O(m·t)	O(m)

**Table 2:** Theoretical performance analysis of private decision-tree-classification protocols. The column “Communication” is the bandwidth complexity of the decision tree. The column “Nodes” is the quantity of nodes running the comparison algorithm in the evaluation.

Scheme	Rounds	Tools	Communication	Nodes	Leakage
[14]	≈5	HE+GC	$O(2^{d})$	*d*	m,d
[28]	≈4	HE,GC	$O(2^{d})$	*d*	m,d
[15]	≥6	FHE,SHE	$O(2^{d})$	*m*	*m*
[16]	6	HE,OT	$O(2^{d})$	*m*	*m*
[11]	4	HE	$O(2^{d})$	*m*	*m*
[29]	≈9	SS	$O(2^{d})$	*m*	m,d
[22]	O(d)	GC,OT	$O(2^{d})$	*d*	m,d
[20]	1	FHE/SHE	$O(2^{d})$	*m*	*m*
[24]	2d−1	FHE/SHE	O(dnt)	*d*	m,d
[25]	8d	FHE/SHE	O(dn)	*d*	m,d
Ours	1	FHE	O(m)	*m*	*m*

**Table 3:** Comparison of the efficiency and bandwidth of the basic operations between the BFV scheme and Lifed-ElGamal scheme protocol. Lifed-ElGamal has no relinearization key and does not support multiplication between ciphertexts. We set “N.A.” for this case. The column “CT” is the bandwidth of the ciphertext. The column “CM/CA” is the runtime for multiplication and addition between ciphertexts. The column “PM/PA” is the time for multiplication and addition between ciphertext and plaintext.

Name	sk/pk/rlk (kb)	CT (kb)	Enc/Dec (ms)	CM/CA (ms)	PM/PA (ms)
BFV	0.144/0.112/0.72	0.144	4.682/1.65	22.251/0.352	0.121/0.002
Lifed-ElGamal	0.584/0.464/N.A.	0.384	0.027/18.879	N.A./0.001	0.021/0.001

**Table 4:** Runtime and communication costs in real datasets for private decision-tree-evaluation protocols. The column “*n*” denotes the dimension of vector, “*d*” denotes the depth of the decision tree, and “*m*” denotes the number of decision nodes in the decision tree. These pieces of information represent the complexity of the decision tree. The column $T_{s}$ is the runtime of the server, and $T_{c}$ is the runtime of the client. Finally, *B* is the communication cost of evaluation.

Dataset	*n*	*d*	*m*	Scheme	$T_{c}$/s	$T_{s}$/s	*B*/kb
Heart Disease	13	3	5	[11]	0.237	0.001	46.848
				Ours	0.224	2.868	9.648
Breast Cancer	9	8	12	[11]	0.523	0.001	139.392
				Ours	0.674	9.227	27.926
Spambase	57	27	58	[11]	4.034	0.015	1530.240
				Ours	6.631	112.795	263.520

## Data Availability

Not applicable.
